# Chromosomal microarray analysis reveals the genetic etiology spectrum of pregnancy loss in 849 products of conception samples

**DOI:** 10.3389/fgene.2026.1980892

**Published:** 2026-10-30

**Authors:** Ming-Xian Kang, Le-Qi Tong, Jing Zhang, Zhao-Hui Wang, Xue Ma, Jia-En Liu, Hua-Ying Hu, Tian-Ying Wei

**Affiliations:** 1 Beijing Jiaen Hospital, Heen Life Medical Research Institute, Beijing, China; 2 McGill University, Montreal, QC, Canada; 3 Prenatal Diagnosis Center, Shijiazhuang Obstetrics and Gynecology Hospital, Hebei Key Laboratory of Maternal and Fetal Medicine, Shijiazhuang, Hebei, China; 4 Medical Innovation Research Division of Chinese PLA General Hospital, Beijing, China

**Keywords:** chromosomal abnormalities, chromosomal microarray analysis, copy number variant, pregnancy loss, products of conception, spontaneous abortion

## Abstract

Objective: To characterize the spectrum and distribution of chromosomal abnormalities detected by chromosomal microarray analysis (CMA) in products of conception (POC) from pregnancy loss, and to assess the association between maternal age and the detection rate of these abnormalities, providing evidence for genetic counseling and recurrence risk assessment. Methods: A total of 852 POC samples from women with pregnancy loss were received at Beijing Jiaen Hospital from January 2016 to December 2025; 849 were successfully analyzed using the Affymetrix CytoScan 750K array following the manufacturer’s standard protocols, while 3 samples were cancelled because immersion in formalin or alcohol had rendered the DNA unsuitable for testing. All detected copy number variants (CNVs) were evaluated for pathogenicity according to ACMG/ClinGen guidelines through comprehensive database queries. The types, frequencies, and distribution characteristics of chromosomal abnormalities were summarized descriptively, and the association between maternal age and the detection rate of pathogenic abnormalities was assessed using the chi-square test, the Cochran–Armitage test for trend, and univariable logistic regression. Results: Abnormalities were detected in 62.19% (528/849) of samples, with numerical abnormalities—trisomies, double trisomies, monosomies, and polyploidies (47 triploid and 6 tetraploid cases)—accounting for the vast majority (82.58% of abnormal samples, 436/528). Trisomy 16 and trisomy 22 were the most prevalent, followed by monosomy X. The remaining 92 samples included other aneuploid abnormalities (n = 35), CNVs (n = 52), and complete hydatidiform moles (genome-wide uniparental disomy, n = 5). The detection rate of pathogenic abnormalities increased with maternal age, from 48.99% in women aged <30 years to 73.81% in women aged ≥40 years (*P* for trend <0.001), with the ≥40-year group showing significantly elevated odds relative to the <30-year group (odds ratio = 2.93, 95% confidence interval: 1.76–4.89). Conclusion: Chromosomal or genomic abnormalities detectable by CMA were identified in 62.19% of the 849 POC samples analyzed. Because CMA does not require cell culture and can detect submicroscopic CNVs, loss of heterozygosity, and uniparental disomy, it is well suited for the genetic evaluation of POC; however, the incremental diagnostic yield of CMA over conventional karyotyping was not assessed in this study.

## Introduction

Spontaneous abortion (SA), defined as the spontaneous loss of a clinically recognized pregnancy before fetal viability (Quenby et al., 2021; American College of Obstetri cians and Gynecologists' Committee on Practice Bulletins—Gynecology, 2018), is the most common complication of early pregnancy. Global estimates indicate that miscarriages occur in approximately 15% of all pregnancies, with an estimated 23 million miscarriages occurring annually worldwide (Quenby et al., 2021). Recurrent spontaneous abortion (RSA), defined as two or more consecutive pregnancy losses (Practice Committee of the American Society for Reproductive Medicine, 2012; The ESHRE Guideline Group on RPL et al., 2018), affects approximately 1% of women in their lifetime. The incidence of pregnancy loss increases significantly with maternal age: women aged 45 years and above experience a 53% miscarriage rate compared to 10% for those aged 25–29 years (Magnus et al., 2019). Despite its substantial physical, psychological, and economic burden on affected families, miscarriage has historically received insufficient attention from both the medical research community and healthcare policymakers (Quenby et al., 2021).

The etiology of pregnancy loss is complex and multifaceted, encompassing genetic abnormalities, anatomical factors, endocrine dysfunction, immune disorders, thrombophilic conditions, and environmental exposures (Practice Committee of the American Society for Reproductive Medicine, 2012). Among these, genetic abnormalities—particularly chromosomal aberrations in the products of conception (POC)—represent the most frequently identified cause. Accumulating evidence suggests that approximately 50%–60% of pregnancy failures can be attributed to genetic abnormalities, primarily involving chromosome numerical abnormalities and copy number variants (CNVs) (Levy et al., 2014). A recent comprehensive review reported that approximately 60% of examined POC harbor chromosomal abnormalities and CNVs (Sahoo et al., 2017). These include aneuploidies, polyploidies, partial monosomies and trisomies, microdeletions and microduplications, as well as complex structural rearrangements. Notably, certain submicroscopic chromosomal imbalances—including microdeletions and microduplications—have been increasingly recognized as important contributors to pregnancy loss, as they may disrupt genes critical for embryonic development and implantation pathways (Wang et al., 2020).

Traditional cytogenetic techniques for analyzing POC have included conventional G-banded karyotyping and fluorescence *in situ* hybridization (FISH). While karyotyping has long served as the standard method for chromosomal evaluation, it requires viable cells for culture, a condition often difficult to achieve with POC tissue. Consequently, culture failure rates remain substantial, limiting the diagnostic yield. Moreover, conventional karyotyping has limited resolution (typically 5–10 Mb) and cannot reliably detect submicroscopic imbalances, such as microdeletions and microduplications, which fall below the detection threshold. FISH, while offering rapid results, is targeted to specific chromosomal regions and lacks genome-wide coverage. These limitations have underscored the need for higher-resolution, culture-independent technologies (Levy et al., 2014; Sahoo et al., 2017).

Chromosomal microarray analysis (CMA) has emerged as a transformative technology for the genetic evaluation of POC. CMA is a high-resolution whole-genome screening technique that simultaneously detects CNVs and, when combined with single nucleotide polymorphism (SNP) probes, can also identify loss of heterozygosity (LOH) and uniparental disomy (UPD) without requiring cell culture. CMA offers superior diagnostic yield compared to conventional karyotyping, with the capacity to detect submicroscopic deletions and duplications (as small as 50–200 kb) that are invisible to older methods (Sahoo et al., 2017; Dhillon et al., 2014). Importantly, CMA does not rely on viable cell growth, making it particularly well-suited for POC analysis, where tissue quality is often suboptimal (Sahoo et al., 2017; Dhillon et al., 2014). Recent meta-analyses and large cohort studies have confirmed that CMA provides additional clinically significant findings over conventional karyotyping in up to 5%–10% of POC cases with normal karyotypes (Finley et al., 2022). The integration of SNP probes further enables the detection of UPD and regions of homozygosity, which are relevant for identifying complete hydatidiform moles and assessing risks for autosomal recessive disorders (Sahoo et al., 2017). Owing to these advantages, professional societies now recommend CMA as the first-line genetic test for evaluating POC in cases of pregnancy loss (Hillman et al., 2013; Wapner et al., 2012; Society for Maternal-Fetal Medicine et al., 2016).

Given the rapidly evolving landscape of reproductive genetics and the increasing adoption of CMA for POC analysis, there remains a need for large-scale studies characterizing the spectrum and distribution of chromosomal abnormalities in Chinese populations. To date, most large-scale CMA studies on POC have been conducted in Western cohorts, and population-specific data from China remain relatively limited. In this study, we performed CMA on 849 consecutive POC specimens from women who experienced pregnancy loss between 2016 and 2025 using the Affymetrix CytoScan 750K array, which integrates both CNV probes and SNP markers. The objectives of this study were: (1) to elucidate the types and frequencies of chromosomal abnormalities in POC; (2) to characterize the contribution of CMA beyond aneuploidy, including CNVs, polyploidies, and UPD; and (3) to assess the association between maternal age and the detection rate of chromosomal abnormalities in POC, thereby providing evidence-based guidance for genetic counseling and prenatal risk assessment.

## Materials and methods

### Study subjects

A total of 852 POC samples from women with pregnancy loss were received at the Genetics Laboratory of Beijing Jiaen Hospital between January 2016 and December 2025. In this study, “pregnancy loss” is used as an umbrella term encompassing spontaneous abortion (the spontaneous loss of a clinically recognized pregnancy before fetal viability), late pregnancy loss, and stillbirth; because gestational age was recorded only for a subset of the received specimens and could not be systematically analyzed, samples derived from pregnancies beyond 24 weeks of gestation could not be excluded retrospectively and were included. Of the 852 samples received, 3 were cancelled because the tissue had been immersed in formalin or alcohol, which compromises DNA integrity and precludes reliable CMA; the remaining 849 samples were successfully analyzed. The 849 analyzed samples comprised 776 chorionic villi (91.4%), 68 embryonic tissue samples (8.0%), and 5 umbilical cord samples (0.6%). Sample types included chorionic villi, embryonic tissue, or umbilical cord, selected according to gestational age and sample condition: chorionic villi were preferentially collected during the first trimester (<12 weeks), embryonic tissue during the second trimester (13–27 weeks), and umbilical cord or embryonic tissue during the third trimester (>28 weeks). Because the testing was performed at the hospital’s internal laboratory and maternal clinical information was not systematically available in the laboratory records, detailed clinical data—including history of gestational infection or exposure to teratogenic substances—could not be retrieved, and no cases were excluded on these clinical grounds. Conventional karyotyping was not performed on any of the included POC samples. The study protocol was approved by the Ethics Committee of our hospital (Approval No. BJH-2026003). All research involving human participants complied with the ethical standards of the 2013 Declaration of Helsinki.

### DNA extraction

POC samples were washed, homogenized, and resuspended in 200 μL of 1×PBS buffer. Whole-genomic DNA was extracted following the standard protocol of the QIAamp Fast DNA Tissue Kit (QIAGEN). The integrity of extracted genomic DNA was verified by 0.75% agarose gel electrophoresis (requiring fragments >20 kb), and DNA concentration was measured using the Qubit dsDNA HS Assay Kit (Thermo Fisher Scientific), with a minimum threshold of 50 ng/μL.

### Chromosomal microarray analysis

The Affymetrix CytoScan 750K array, containing 550,000 copy number marker probes and 200,000 SNP probes, was used for copy number detection, with simultaneous analysis of LOH and UPD. Using the matching reagents and instruments, genomic DNA from POC samples was processed through digestion, ligation, and amplification according to Affymetrix’s standard operating procedures. The amplified products were purified, fragmented, and labeled. Labeled samples were hybridized to the 750K array at 50 °C with rotation at 60 rpm. Hybridized arrays were washed, stained, and scanned, and the data were analyzed using ChAS software with the human genome GRCh38 (hg38) as the reference sequence.

### Data analysis

CNVs identified by ChAS software were evaluated for pathogenicity according to the guidelines of the American College of Medical Genetics and Genomics (ACMG) (Spurdle et al., 2023; Riggs et al., 2020), supplemented by comprehensive queries of the following international genomic databases and relevant literature: ClinVar, DECIPHER, UCSC, Online Mendelian Inheritance in Man (OMIM), International Standards for Cytogenomic Arrays (ISCA), and Database of Genomic Variants (DGV). CMA results were interpreted using the following predefined categories: an abnormal result was defined as the detection of a segment deletion ≥200 kb, a segment duplication ≥500 kb, or LOH ≥10 Mb within chromosomal arms or ≥5 Mb at terminal regions within the reportable range; detected CNVs were classified as pathogenic, likely pathogenic, variant of uncertain significance (VUS), likely benign, or benign; and a negative result indicates only that no abnormality was detectable within this reportable range, not a genetically normal specimen.

### Statistical analysis

A systematic quantitative descriptive approach was employed to statistically characterize the types, frequencies, and distribution patterns of chromosomal abnormalities, without distinguishing the sex of the POC samples. The statistical framework included: (1) Typological analysis: establishing a classification system comprising numerical abnormalities (aneuploidy), polyploidies (triploidy and tetraploidy), CNVs, and complete hydatidiform moles; (2) Frequency calculation: using event rates (n/N×100%) to quantify the occurrence frequencies of different categories and subtypes, with the denominator stated explicitly for each analysis; and (3) Distribution pattern analysis: comparing frequencies and proportions across type categories, supplemented by detailed subgroup analyses. To formally assess the association between maternal age and the detection rate of pathogenic CMA abnormalities, three complementary analyses were performed: (1) the overall chi-square test across the four evenly spaced 5-year maternal age groups (<30, 30–34, 35–39, and ≥40 years); (2) the Cochran–Armitage test for trend using ordinal age-group scores; and (3) univariable binary logistic regression, reported as odds ratios (ORs) with 95% confidence intervals (CIs), both per age-group increment and for each age group relative to the <30-year reference group. All tests were two-sided, and *P* < 0.05 was considered statistically significant. Because these analyses were based on aggregated group-level data, adjustment for potential confounding variables was not possible.

## Results

### Overview of detection results

The sample inclusion and analysis process is summarized in Figure 1. Representative normal and abnormal CMA results are shown in Supplementary Figure S1 and Figure 2, respectively. Of the 849 POC samples analyzed, 321 (37.81%, 321/849) yielded negative results, defined as no abnormality detected within the reportable range by high-resolution CMA (segment deletions ≥200 kb, segment duplications ≥500 kb, LOH ≥10 Mb within chromosomal arms or ≥5 Mb at terminal regions); as noted above, a negative result does not constitute evidence of a genetically normal specimen. The remaining 528 samples (62.19%, 528/849) were positive, i.e., harbored abnormalities detectable within the reportable range. To avoid double counting, the 528 positive samples were assigned to mutually exclusive categories as follows: 312 trisomies (59.09% of positive samples), 16 double trisomies (3.03%), 55 monosomies (10.42%), 35 other aneuploid abnormalities (6.63%), 53 polyploidies (47 triploid/near-triploid and 6 tetraploid/near-tetraploid cases; 10.04%), 52 CNV results (9.85%), and 5 complete hydatidiform moles with genome-wide UPD (0.95%). The proportions of each category relative to the total sample size (n = 849) are shown in Figure 3. The 35 other aneuploid abnormalities (6.63%) comprised cases that combined a whole-chromosome aneuploidy with additional segmental imbalances, such as microduplications or copy-neutral LOH (e.g., trisomy 21 with an X-chromosome microduplication, or trisomy 16 with chromosome 19 LOH), or that involved abnormalities of three or more chromosomes, and therefore did not fit any of the mutually exclusive categories above.

**FIGURE 1:**
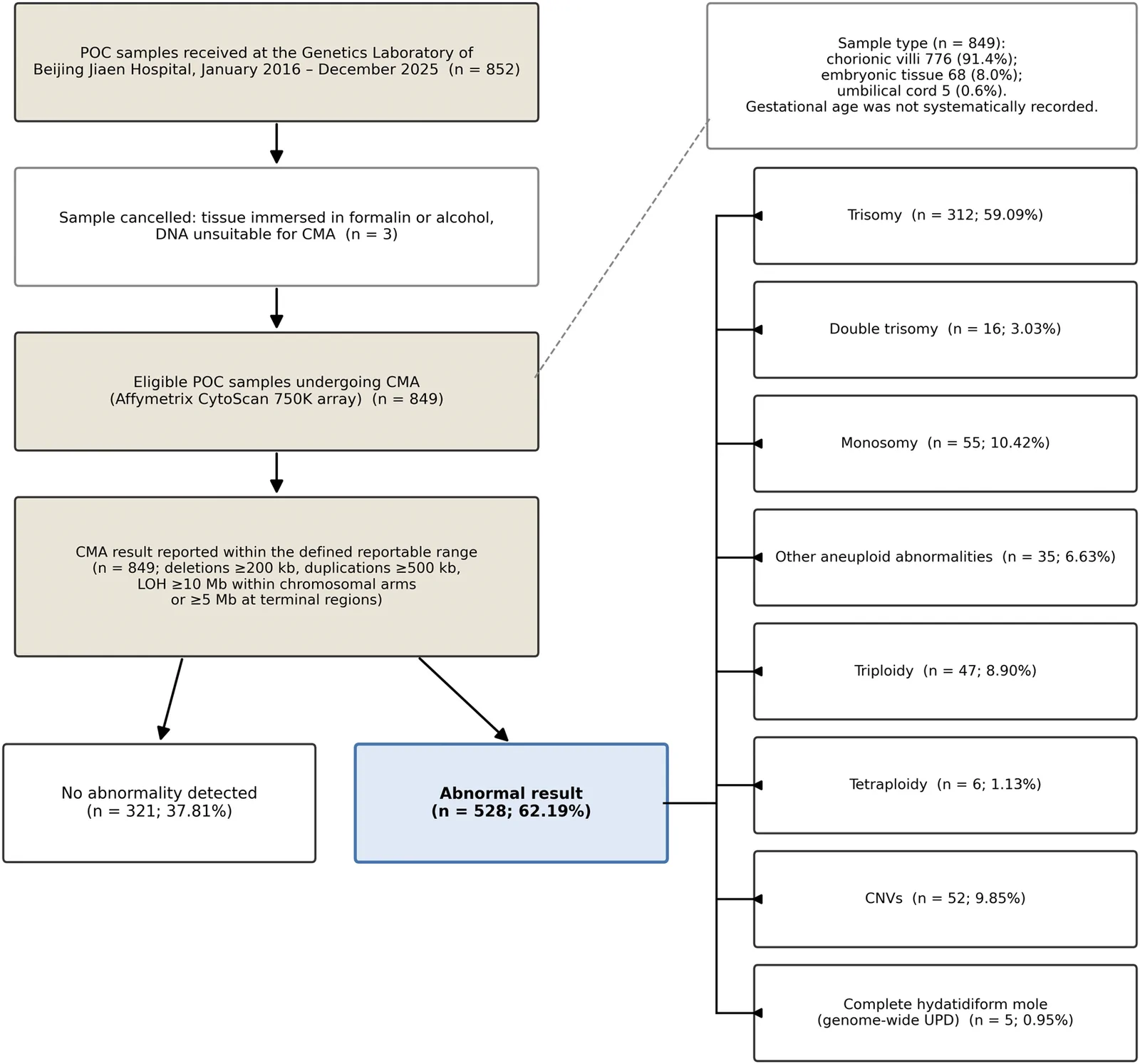
Flowchart of sample inclusion and analysis. Of 852 POC samples received at the study laboratory, 3 were cancelled because immersion in formalin or alcohol rendered the DNA unsuitable for CMA; the remaining 849 eligible samples underwent CMA on the Affymetrix CytoScan 750K array, and results were classified as no abnormality detected or abnormal, with abnormal samples subdivided into mutually exclusive categories. The flowchart also shows the distribution of sample types (chorionic villi, n = 776 [91.4%]; embryonic tissue, n = 68 [8.0%]; umbilical cord, n = 5 [0.6%]). Gestational age was not systematically recorded; a breakdown by gestational age was therefore not possible.

**FIGURE 2:**
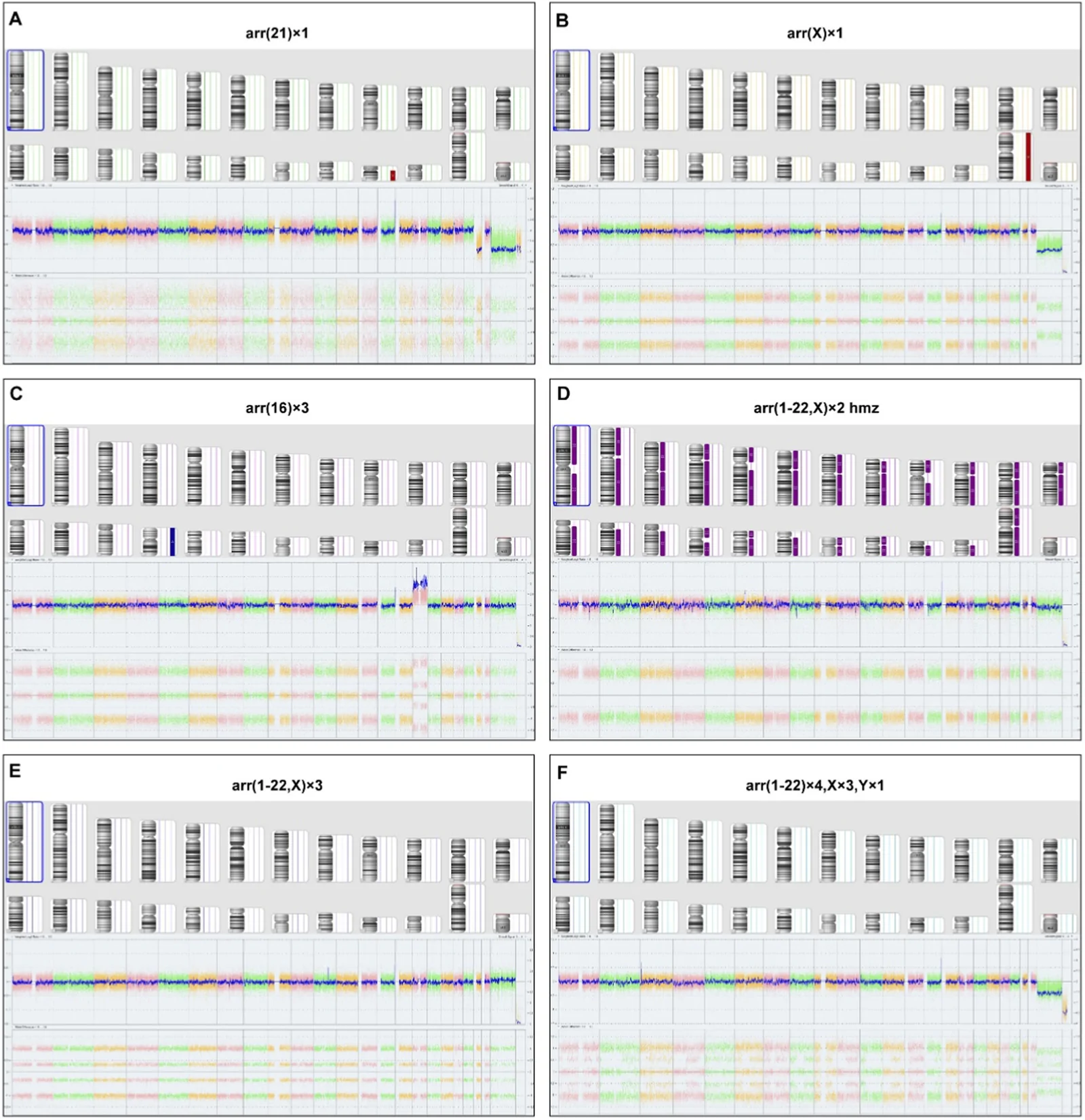
Abnormal chromosomal microarray analysis results. **(A)** arr(21)×1. High-resolution CMA of male POC tissue detected monosomy 21. **(B)** arr(X)×1. High-resolution CMA of female POC tissue revealed monosomy X. **(C)** arr(16)×3. High-resolution CMA of female POC tissue identified trisomy 16, the most frequently observed chromosomal trisomy in this study. **(D)** arr(1-22,X)×2 hmz. High-resolution CMA of female POC tissue showed features consistent with UPD, indicative of a complete hydatidiform mole. **(E)** arr(1-22,X)×3. High-resolution CMA indicated a female triploid embryo in the POC sample. **(F)** arr(1-22)×4,X×3,Y×1. High-resolution CMA revealed a male tetraploid embryo in the POC sample.

**FIGURE 3:**
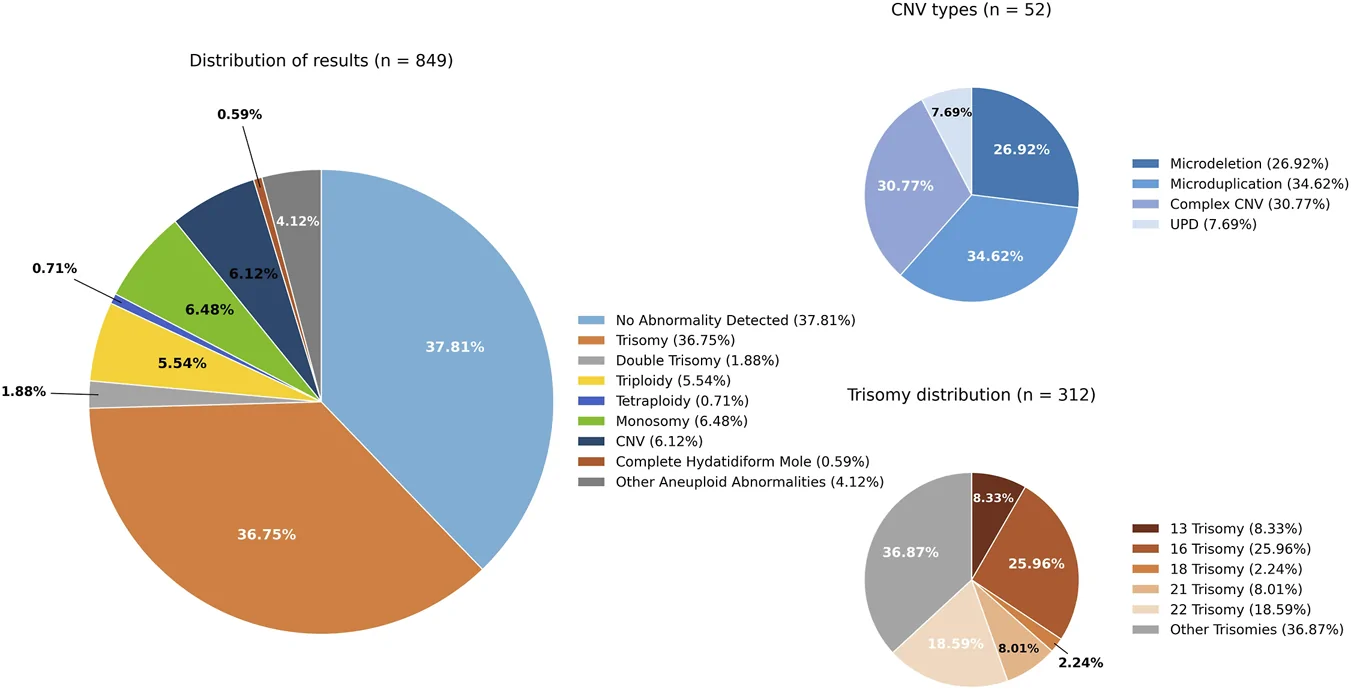
Distribution of chromosomal abnormalities. The left panel depicts the distribution of all results (n = 849), including cases with no abnormality detected; the upper right panel shows the classification of CNV types (n = 52); and the lower right panel presents the distribution of trisomies (n = 312).

### Chromosomal numerical abnormalities

The distribution of chromosomal numerical abnormalities is shown in Figure 4. Among the 312 trisomy samples (the sum of all samples before the dashed line in Figure 4), trisomy 16 accounted for 81 cases (25.96%), followed by trisomy 22 (58 cases, 18.59%), trisomy 13 (26 cases, 8.33%), trisomy 21 (25 cases, 8.01%), and trisomy 18 (7 cases, 2.24%), with other chromosomal trisomies accounting for 115 cases (36.87%). Among the 55 monosomy samples, 54 (98.18%) were monosomy X and 1 (1.82%) was monosomy 21. Mosaic aneuploidies were also observed (n = 10): 8 mosaic trisomies, involving chromosomes 15, 3, 8, 13, 9, 2, 12, and 22, and 2 mosaic monosomies, involving chromosomes X and 21. In addition, one mosaic CNV (a partial mosaic duplication of chromosome 7q) was detected (Table 1; Supplementary Table S1).

**FIGURE 4:**
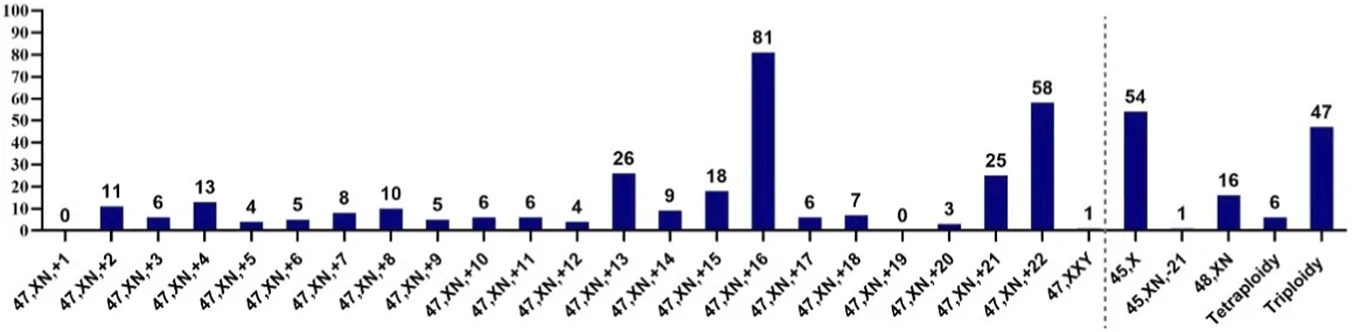
Distribution of chromosomal numerical abnormalities. Trisomies are arranged in ascending chromosome order (47,XN,+1 through 47,XN,+22, followed by 47,XXY). The dashed line separates the trisomies from the monosomies (45,X and 45,XN,-21), the double trisomies (48,XN), and the polyploidies (tetraploidy and triploidy).

**TABLE 1:** Summary of copy number variants (CNVs) detected by chromosomal microarray analysis (n = 52).

CNV type	n	Proportion of 52 CNV results
Microdeletion	14	26.92%
Microduplication	18	34.62%
Complex CNV	16	30.77%
UPD (copy-neutral LOH)	4	7.69%
Total	52	100.00%
ACMG/ClinGen classification	n	Proportion of 52 CNV results
Pathogenic	21	40.38%
Likely pathogenic	5	9.62%
Variant of uncertain significance (VUS)	21	40.38%
Likely benign	3	5.77%
Benign	2	3.85%
Pathogenic + likely pathogenic	26	50.00%

The case-by-case list with gene-level annotations and ACMG/ClinGen classifications is provided in Supplementary Table S1.

### Polyploid abnormalities and complete hydatidiform moles

A total of 53 polyploid abnormalities (10.04% of the 528 positive samples) were identified, including 47 triploid/near-triploid cases (which can give rise to partial hydatidiform moles) and 6 tetraploid/near-tetraploid cases. Additionally, 5 complete hydatidiform moles (0.95% of positive samples) were detected, characterized by genome-wide uniparental disomy (UPD).

### Microdeletions, microduplications, and UPD (CNVs)

A total of 52 CNV results were identified, comprising 26.92% microdeletions, 34.62% microduplications, 30.77% complex variants, and 7.69% UPD, as shown in the upper right panel of Figure 3. Of note, UPD detected by CMA manifests as copy-neutral LOH within the affected region(s), and the parental origin of the UPD was not confirmed in this study. To date, no evidence has been found linking single-chromosome or partial-chromosome UPD to pregnancy loss, although its presence may increase the risk of recessive genetic disorders in the embryo.

The detected CNVs were classified into five categories according to the ACMG/ClinGen pathogenicity assessment guidelines (Spurdle et al., 2023; Riggs et al., 2020): pathogenic, likely pathogenic, variants of uncertain significance (VUS), likely benign, and benign. Among the 52 cases with detected CNVs or CNV-containing results, 21 were pathogenic, 5 were likely pathogenic, 21 were VUS, 3 were likely benign, and 2 were benign. Pathogenic and likely pathogenic CNVs combined totaled 26 cases, accounting for 50.00% (26/52) of all CNVs. The detected CNVs involved multiple chromosomes; detailed CNV information is presented in Table 1, and the corresponding gene-level annotations are provided in Supplementary Table S1.

### Chromosomal abnormalities and maternal age

To further investigate the association between maternal age and pathogenic CMA results, subjects were divided into four evenly spaced 5-year age groups: <30, 30–34, 35–39, and ≥40 years. Table 2 summarizes the proportions of cases with pathogenic variants (including chromosomal numerical abnormalities and pathogenic/likely pathogenic CNVs) detected in each group. The detection rate increased from 48.99% (73/149) in the <30-year group to 55.81% (168/301) in the 30–34-year group, 61.54% (168/273) in the 35–39-year group, and 73.81% (93/126) in the ≥40-year group. The difference across groups was statistically significant (chi-square = 19.60, *df* = 3, *P* < 0.001), and the Cochran–Armitage test showed a significant linear trend with increasing age (*z* = 4.33, *P* < 0.001). In univariable logistic regression, the odds of a pathogenic result increased 1.38-fold per age-group increment (95% CI: 1.19–1.61; *P* < 0.001); compared with the <30-year reference group, the ORs were 1.32 (95% CI: 0.89–1.95; *P* = 0.173) for 30–34 years, 1.67 (95% CI: 1.11–2.49; *P* = 0.013) for 35–39 years, and 2.93 (95% CI: 1.76–4.89; *P* < 0.001) for ≥40 years. As these analyses were unadjusted for potential confounders, this association should be interpreted as an observed relationship rather than evidence of an independent effect of maternal age.

**TABLE 2:** Association between maternal age and the detection rate of pathogenic chromosomal abnormalities in 849 products of conception samples.

Maternal age (years)	Total cases	Pathogenic cases	Abnormality rate	Odds ratio (95% CI)	*P* Value
<30	149	73	48.99%	Reference	—
30–34	301	168	55.81%	1.32 (0.89–1.95)	0.173
35–39	273	168	61.54%	1.67 (1.11–2.49)	0.013
≥40	126	93	73.81%	2.93 (1.76–4.89)	<0.001

“Pathogenic cases” includes only pathogenic and likely pathogenic findings (chromosomal numerical abnormalities, other aneuploid abnormalities, complete hydatidiform moles, and pathogenic/likely pathogenic copy number variants (CNVs)), excluding variants of uncertain significance (VUS), likely benign, and benign CNVs; the total (502) therefore differs from the overall number of positive results (528). Overall chi-square test: χ^2^ = 19.60, *df* = 3, *P* < 0.001; Cochran–Armitage test for trend: *z* = 4.33, *P* < 0.001; univariable logistic regression per age-group increment: OR, 1.38 (95% CI: 1.19–1.61), *P* < 0.001. Odds ratios are unadjusted.

## Discussion

This study characterized the spectrum and distribution of chromosomal abnormalities detected by CMA in 849 POC samples from a single Chinese center. The principal finding is that chromosomal or genomic abnormalities detectable by CMA were identified in 62.19% of the analyzed samples, consistent with previous reports that approximately 50%–60% of pregnancy losses are attributable to chromosomal abnormalities (Levy et al., 2014; Sahoo et al., 2017; Zhuang et al., 2022). The observed spectrum was dominated by numerical abnormalities, including polyploidies, which accounted for 82.58% of abnormal results, with trisomy 16 (25.96% of trisomies), trisomy 22 (18.59%)—possibly related to the meiotic error-prone nature of the telomeric region of its short arm (22pter) (Trevisan et al., 2024)—and monosomy X (54 of 55 monosomies) predominating. The high frequency of trisomy 16 is in line with its postulated biological characteristics, including rearrangement-prone pericentric regions and the dosage sensitivity of key developmental genes such as SOX8 and TSC2 (Parinayok et al., 2022), whereas the predominance of monosomy X likely reflects the roles of X-linked dosage-sensitive genes such as SHOX and XIST in early embryonic survival (Yoon et al., 2023). Conversely, trisomies of chromosomes 1 and 19 are only rarely observed, presumably because the low probability of meiotic nondisjunction of chromosome 1 and the severe dosage imbalance caused by an extra copy of chromosome 19—the chromosome with the highest gene density—arrest development at a very early stage (Gu et al., 2021; Grimwood et al., 2004).

Relative to previous large cohorts, the abnormality detection rate of 62.19% in the present study lies at the high end of the published range: the largest single-laboratory CMA series reported clinically significant abnormalities in 53.7% of 7,396 successful results (Sahoo et al., 2017), a systematic review of 5,507 early pregnancy losses estimated the incremental yield of CMA over karyotyping at only 2%–4% (Pauta et al., 2018), and Chinese cohorts have reported broadly comparable detection rates (Zhuang et al., 2022; Gu et al., 2021; Xu et al., 2024). The spectrum observed here—predominance of numerical abnormalities, including polyploidies (82.58% of abnormal results), led by trisomy 16, trisomy 22, and monosomy X—is consistent with these previous reports (Sahoo et al., 2017; Finley et al., 2022; Zhuang et al., 2022). The contribution of the present study therefore lies not in the method itself, which is an established best practice for the evaluation of pregnancy loss (Society for Maternal-Fetal Medicine et al., 2016), but in the following aspects: (1) it is among the largest single-center Chinese cohorts analyzed on a single platform (CytoScan 750K) over a 10-year period (2016–2025); (2) it provides a formal statistical assessment of the association with maternal age using a trend test and logistic regression; and (3) it integrates UPD/homozygosity analysis, enabling identification of complete hydatidiform moles that cannot be detected by conventional karyotyping.

Beyond aneuploidy, CMA contributed additional clinically relevant information. It identified 53 polyploidies, 52 CNV results (of which 50.00% were pathogenic or likely pathogenic), and 5 cases of genome-wide UPD indicative of complete hydatidiform moles. Triploidy most commonly arises from fertilization of a haploid egg by two sperm or by a diploid sperm, and tetraploidy is thought to result mainly from failure of the first zygotic division or from fertilization of a polar-body-retaining egg (Massalska et al., 2021; Yildirim et al., 2023). The detection of complete moles has direct clinical implications, given the 18%–29% risk of gestational trophoblastic neoplasia (Masset et al., 2021), and is uniquely enabled by the SNP-probe content of the array, as conventional karyotyping and FISH cannot detect UPD. CNVs may exert their effects through gene dosage (Zhuang et al., 2020), and structural abnormalities such as microdeletions and microduplications are likewise key factors in pregnancy loss (Nikitina et al., 2020), although the relationship between the number of miscarriages and the rate of embryonic chromosomal abnormalities remains controversial (Sheng et al., 2021). Unbalanced CNVs accounted for 17.31% (9/52) of the observed copy number abnormalities and may originate from balanced parental rearrangements, supporting current recommendations for karyotyping of both partners in indicated cases (Kearney et al., 2011; South et al., 2013). The relatively high proportion of VUS (21/52) underscores the continuing challenge of interpretation and the need for reclassification as databases mature (Dimova et al., 2020).

Higher maternal age was associated with a higher prevalence of detectable abnormalities, with the detection rate increasing from 48.99% in women aged <30 years to 73.81% in women aged ≥40 years (*P* for trend <0.001). This observation is biologically plausible, as the probability of chromosomal nondisjunction during meiosis increases with oocyte age (Nagaoka et al., 2012). However, causality and clinical predictive value cannot be inferred from the present study: the analysis was unadjusted for potential confounding variables, and the detection rate of nearly half of the samples in the youngest group indicates that genetic risk is by no means confined to advanced maternal age. These findings therefore support, rather than mandate, the consideration of high-resolution genomic testing such as CMA in the evaluation of pregnancy loss across all age groups (Wapner et al., 2012).

Several limitations should be considered when interpreting these results. First, this was a single-center cohort, which introduces the possibility of selection bias; in addition, selection bias cannot be excluded, as it is unclear whether the 849 cases represent all pregnancy losses managed at the institution or a selected subset in whom CMA testing was performed, and the patient mix of the hospital may not be fully representative of the general population. Second, there was no control group of pregnancies with normal outcomes, limiting the interpretation of the observed spectrum. Third, conventional karyotyping was not performed on any of the included POC samples; consequently, the concordance between methods, the proportion of abnormalities detectable by conventional cytogenetics, and the incremental diagnostic yield of CMA could not be quantified. Fourth, maternal cell contamination, a well-recognized source of misclassification in POC studies, was not systematically assessed and should be explicitly evaluated in future studies; relatedly, the detection of low-level mosaicism is limited by the probe density and analytical thresholds of the platform. Fifth, a negative result in this study indicates only the absence of an abnormality detectable within the defined reportable range (deletions ≥200 kb, duplications ≥500 kb, and specified LOH thresholds) and should not be interpreted as evidence of a genetically normal specimen; CMA also cannot detect balanced rearrangements, point mutations, or very small imbalances. Finally, the maternal-age analysis was limited by the scarcity of additional clinical variables (e.g., BMI, smoking, reproductive history, and gestational age at loss), which restricted the ability to explore potential confounding factors such as endocrine, infectious, environmental, and thrombophilic conditions (Li et al., 2021), and the inclusion of samples from pregnancies beyond 24 weeks means that the cohort encompasses a heterogeneous gestational-age spectrum that may differ in its abnormality distribution.

## Data Availability

The datasets for this article are not publicly available due to concerns regarding participant/patient anonymity. Requests to access the datasets should be directed to the corresponding author upon reasonable request.
